# Isolation and Characterization of Thermophilic Bacteria from Jordanian Hot Springs:* Bacillus licheniformis* and* Thermomonas hydrothermalis* Isolates as Potential Producers of Thermostable Enzymes

**DOI:** 10.1155/2017/6943952

**Published:** 2017-10-15

**Authors:** Balsam T. Mohammad, Hala I. Al Daghistani, Atef Jaouani, Saleh Abdel-Latif, Christian Kennes

**Affiliations:** ^1^Pharmaceutical and Chemical Engineering Department, School of Applied Medical Sciences, German Jordanian University, P.O. Box 35247, Amman 11180, Jordan; ^2^Department of Medical Laboratory Sciences, College of Science, Al Balqa Applied University, As-Salt, Jordan; ^3^Institut Supérieur des Sciences Biologiques Appliquées de Tunis, Faculté des Sciences de Tunis, Laboratoire Microorganismes et Biomolécules Actives LR03ES03, Université de Tunis El Manar, 2092 Tunis, Tunisia; ^4^Chemical Engineering Laboratory, Faculty of Sciences and Center for Advanced Scientific Research (CICA), University of A Coruña, 15008 A Coruña, Spain

## Abstract

The aim of this study was the isolation and characterization of thermophilic bacteria from hot springs in Jordan. Ten isolates were characterized by morphological, microscopic, biochemical, molecular, and physiological characteristics. Sequencing of the 16S rDNA of the isolates followed by BLAST search revealed that nine strains could be identified as* Bacillus licheniformis* and one isolate as* Thermomonas hydrothermalis*. This is the first report on the isolation of* Thermomonas* species from Jordanian hot springs. The isolates showed an ability to produce some thermostable enzymes such as amylase, protease, cellulose, gelatins, and lecithin. Moreover, the UPGMA dendrogram of the enzymatic characteristics of the ten isolates was constructed; results indicated a high phenotypic diversity, which encourages future studies to explore further industrial and environmental applications.

## 1. Introduction

Geothermal areas considered the source of the main habitats of thermophilic microorganisms [1]. Geothermal features are not common ecological features; they occur in clusters, in a few widely separated locations of the world where the conditions are right for their occurrence. Due to this specific nature of the geothermal sources, hot springs are available in a few areas only. The best recognized areas and those most studied are in Iceland, United States, New Zealand, Japan, Italy, Indonesia, Central America, and Central Africa [2–4]. The attractive feature of hot water resources is the ecology with its variety of the organisms [5] and the molecular strength of its components [6].

Over the last years, extremophiles with its different categories, thermophiles (high temperature), acidophiles (low pH), alkaliphiles (high pH), halophiles (high salinity), and psychrophiles (low temperature) [7], have fascinated researchers in many fields, due to their ability to withstand and function under extreme conditions.

Thermophilic microorganisms (optimum growth temperature of 50°C or above) have attracted great attention among extremophiles because they are sources of thermostable enzymes (such as amylases, cellulases, chitinases, pectinases, xylanases, proteases, lipase, and DNA polymerases); these enzymes show unique features that can be suitable for performing biotechnological processes at elevated temperatures [8]. Moreover, they have been reported to be more stable against many solvents, detergents, and acidic and alkaline pH [9, 10]. Among these commercially important enzymes are the protease enzymes: alkaline protease possesses the property of a great stability when used in detergents and protease enzymes have found applications in bioindustries such as washing powders, food industry, leather processing, and pharmaceuticals and for studies in biology [11, 12]. Moreover, cellulase enzymes showed great commercial potential for the production of glucose feedstock from agricultural cellulosic materials [13] and in the production of bioethanol and value-added organic compounds from renewable agricultural residues [14]. Various enzymes have significance in applications in bioindustries; for example, protease and amylases are used together in many industries such as the food industry, detergent industries, and pharmaceuticals [15]. Another important enzyme gelatinase has gained an importance as targets for drug developments, because of their role in connective tissue degradation linked with tumor metastasis [16].

Thermophiles can be categorized into moderate thermophiles (growth optimum, 50–60°C), extreme thermophiles (growth optimum, 60–80°C), and hyperthermophiles (growth optimum, 80–110°C) [17]. Thermophiles have been isolated from different ecological zones (e.g., hot springs and deep sea) of the earth. The organisms with the highest growth temperatures (103–110°C) are members of the genera* Pyrobaculum*,* Pyrodictium*,* Pyrococcus*, and* Melanopyrus *belonging to Archaea; within Fungi, the Ascomycetes and Zygomycetes classes have high growth temperatures [18], while, in case of bacteria,* Thermotoga maritime* and* Aquifex pyrophilus* exhibit the highest growth temperatures of 90 and 95°C, respectively [19]. Thermophilic microorganisms can be classified as Gram-positive or Gram-negative, they can exist under aerobic or anaerobic conditions, and some of them can form spores. Due to their increased importance, potential applications, and roles in different fields, scientists have concentrated their studies to discover new genus and species across the world [20–22].

In Jordan, many hot springs are available in different regions across the country, with temperature ranging between 30 and 63°C. Their detailed distribution and characteristics have been described in the literature [23]. There are about 200 thermal springs in Jordan spread across the country. Among the major sites are Al Hammah springs, North Shouneh well, Zarqa Ma'in wells, Al Azraq springs, Al Barbitah spring, and Afra springs [24].

Although interest in studying thermophiles from hot springs in Jordan has been demonstrated by a few previous microbial studies, still no sustained research had focused on further utilization of these thermophiles. The aim of the present study is to establish a continuous research line for screening, isolation, and characterization of new extremophilic microorganisms that can possess high biotechnological and environmental potential.

## 2. Material and Methods

### 2.1. Samples Collection and Characterization

A total of twenty water samples were collected from hot springs using 200 ml sterile thermal glass containers. Five hot springs in Jordan were chosen to collect the sample, including Hammamat Ma'in, Zara Dead Sea, Hammamat Afra, Al-Burbita, and Al-Hemma. Three replicates were taken for each sample from the same spot of the same location and three different depths were considered including 10 cm, 20 cm, and 30 cm. The temperature, pH, and electrical conductivity were measured by using Thermometer, pH meter and Conductometer, respectively. Water samples were used immediately for enrichment in nutrient broth at 55°C. One-day enrichment culture was streaked on nutrient agar and blood agar base (HiMedia, Mumbai) to obtain separate colonies. Plastic freezing bags were used to avoid drying of the samples during incubation. Characterization of each isolate was done by examination of colony color, size, elevation, margin, texture, pigmentation, and hemolysis on 5% sheep red blood agar media, in addition to Gram staining.

### 2.2. Genotypic Study

#### 2.2.1. ITS-PCR Fingerprinting Transcribed Spacers

Genomic DNA from pure strains is extracted using a DNA extraction kit (Promega kit). DNA concentrations were measured by using Nanodrop Spectrophotometer (Bio-Rad). Amplification of the 16S–23S internal transcribed spacer region (ITS) was performed as previously described [25] using, respectively, the universal primers S-D-Bact-1494-a-20 (5′GTCGTAACAAGGTAGCCGTA3′) and L-D-Bact-0035- a-15 (5′CAAGGCATCCACCGT 3′). The amplification reaction mixture consists of 1x PCR reaction buffer, 2.5 mM MgCl_2_, 0.2 mM deoxynucleoside triphosphate, 0.3 mM of each primer, 1 U Taq DNA polymerase, and 1 *µ*l of total DNA. The PCR program consists of an initial step at 94°C for 3 min, 35 cycles of denaturation at 94°C for 45 s, annealing for 1 min at 55°C, and elongation for 2 min s at 72°C, followed by final elongation step at 72°C for 8 min. The ITS-PCR amplification patterns are migrated 1% agarose gels in 0.5x Tris borate-EDTA buffer and stained for 30 min in 0.5 mg liter^−1^ ethidium bromide solution. ITS-PCR profiles are compared and the strains showing comparative profiles are considered identical haplotype.

#### 2.2.2. 16S rDNA Amplification and Sequencing

Genomic DNA was extracted and purified according to Sambrook and Russell [26]. Sequencing of 16S rDNA of the isolate and amplification of the target gene were done using the universal bacterial primer 1492R (5′- TAC GGY TAC CTT GTT ACG ACT T-3′) and the domain bacteria-specific primer 27F (5′- AGA GTT TGA TCM TGG CTC AG-3′). Amplification of DNA was carried out under the following conditions: denaturation at 94°C for 5 min followed by 30 cycles of 94°C for 30 s, 52°C for 30 s, 72°C for 1.5 min, and final extension at 72°C for 10 min. Amplified PCR products of bacterial isolates were analyzed by electrophoresis with 1% agarose gel. The PCR product was purified using QIA quick PCR purification kit (Qiagen). The purified PCR products were sequenced by Genewiz Inc., USA, using Genetic Analyzer (Applied Biosystems 3130 XL, Switzerland). The deduced sequence was subjected to BLAST algorithm from the National Centre of Biotechnology, Bethesda, MD, USA to retrieve for homologous sequences in GenBank. Phylogenetic tree was constructed by performing distance matrix analysis using the NT system. Database search and comparisons were done with the BLAST database.

### 2.3. Metabolic and Biochemical Characterization of the Isolates

Haplotypes were tested by API 50CHB strips (BioMerieux, Inc., France) for utilization of the following substrates: glycerol, erythritol, D-arabinose, L-arabinose, ribose, D-xylose, L-xylose, Adonitol, Β-methyl xyloside, galactose, glucose, fructose, mannose, L-sorbose, rhamnose, dulcitol, inositol, sorbitol, mannitol, L-methyl-D-mannoside, D-methyl-D-glucoside, N-acetylglucosamine, amygdalin, arbutin, aesculin, salicin, cellobiose, maltose, lactose, sucrose, Trehalose, gentiobiose, melibiose, raffinose, melezitose, starch, glycogen, inulin, D-turanose, D-tagatose, D-fucose, L-fucose, D-lyxose, D-arabitol, L-arabitol, xylitol, gluconate, and 2,5-ketogluconate. One hundred *µ*l of suspended bacteria was injected into the strips and incubated at 50–55°C for 48 h. Any change in the color to yellow was measured according to kit instructions. The presence of catalase and oxidase enzymes was investigated according to the methods described by Prescott et al. [27].

### 2.4. Assessment of Enzymatic Production

#### 2.4.1. Cellulase Activity

CMC Plate Assay was used for detection of cellulase producing isolates [28]. The plates were incubated at 37°C for 5 days to allow the secretion of cellulase. After incubation, the agar medium was overflown with an aqueous solution of Congo red (1% w/v) for duration of 15 minutes. The Congo red solution was then discarded, and the plates were further treated by flooding with 1 M NaCl for 15 minutes. The formation of clear zone of hydrolysis indicated cellulose degradation as adopted from Shaikh et al. [29]. The colonies with clearance zone were plated in minimal agar medium supplemented with 1% CMC and incubated at 37°C and stored at 4°C for further studies.

#### 2.4.2. Lecithinase Activity

For lecithinase enzyme, 10 ml of the 50% egg yolk was added to 90 ml of sterilized Tryptic soy agar. The formation of a white precipitate around or beneath the inoculum spot revealed lecithinase formation [30].

#### 2.4.3. Lipase Activity

Lipase activity was observed by the appearance of a turbid halo around the inocula on Tryptic soy agar plates supplemented with 1% Tween 80 as explained by Rollof et al. [31].

#### 2.4.4. Protease Activity

Protease activity was detected on Muller-Hinton agar containing 3% skimmed milk. Plates were streaked with test strains followed by incubation at 37°C for 24 h. The presence of a transparent zone around the colonies indicated caseinase activity [32].

#### 2.4.5. Gelatinase Activity

Gelatinase production was detected by stab inoculating the test strain on nutrient agar supplemented with 3% gelatin kept at 37°C for 24 hours followed by refrigeration at 4°C for 30 min. Liquefaction of gelatin was considered positive [33].

#### 2.4.6. Amylase Activity

Starch hydrolysis method was used to identify the amylolytic properties. The starch agar plates were streaked by microbial isolates followed by their incubation at 37°C for 24 hours. After incubation, 1% iodine solution (freshly prepared) was flooded on the starch agar plate. The presence of blue color around the growth indicated negative results [34].

## 3. Results

### 3.1. Characterization of the Samples

Five hot springs sites were prospected in Jordan between September 2015 and March 2016. The hot springs were located in different cities and represent a moderate thermophilic to thermophilic (39.9–60°C) and neutrophilic to alkalophilic (pH 7.03–8.6) environments with variable electrical conductivity (0.51–3.27 ms cm^−1^). Hammamat Ma'in and Zara hot spring were associated with the highest temperature, pH, and conductivity (Table 1).

### 3.2. Morphological and Biochemical Examination of the Isolates

**Figure 1:**
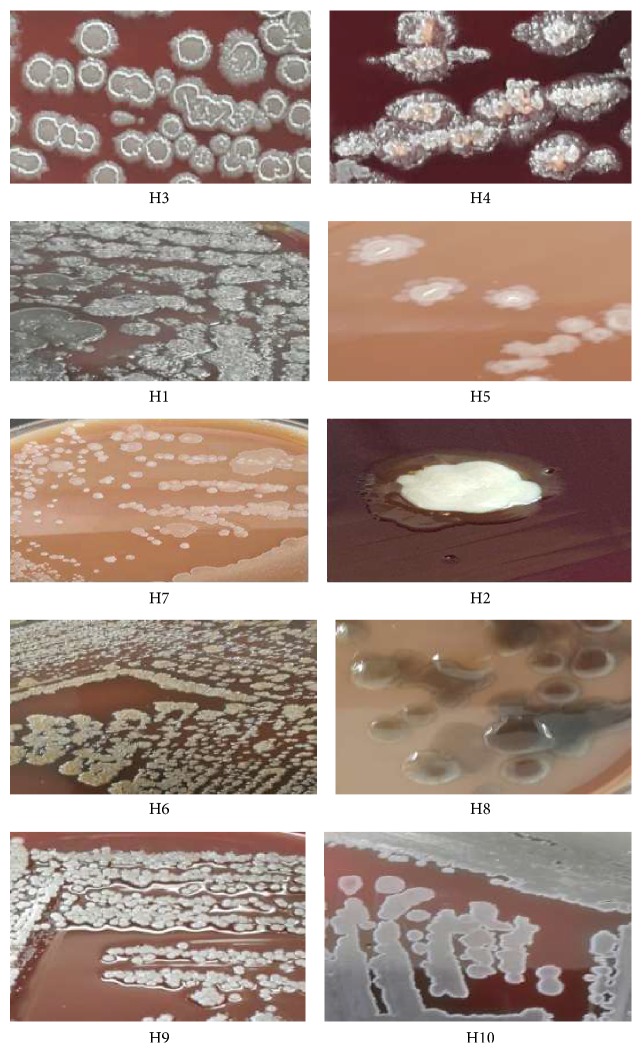
Variation in colonial morphology of bacterial isolates from the hot springs using 5% sheep blood agar.

Various identification tests like endospore formation, motility, anaerobiosis, catalase, and oxidase were performed. Morphological, microscopic observation and biochemical test indicated that the bacteria belonged to the* Bacillus* sp. The selected strains were observed and growth characteristics were studied (Figure 1).

Morphologically, the isolates showed some variation in the color, margin, shape, and texture of the colonies (Table 2). They were grey, creamy, and white; opaque or translucent; rough or smooth; with regular or irregular edges. Colonies might appear finely wrinkled and adherent to the agar surface. They exhibit alpha- or beta-hemolytic activity on 5% sRBCs. Based on Gram staining, the isolates were found mostly to be Gram-positive and microscopic observation revealed spore- forming rod-shaped bacterium arranged in chain. Growth occurred on BHI agar, nutrient agar, and blood agar but not on MacConkey agar. The API 50CHB profiles for the ten isolates demonstrated phenotypic diversity, and none of the ten isolates shared the same phenotypic patterns (Table 3). As one can see, the utilization of carbohydrates varies; for example, strain H4 can utilize a variety of substrates, in comparison to H6 which can utilize only four substrates. Moreover, strain H8 was the only one capable of yielding reaction with Starch and Glycogen. From a taxonomic point of view, strain H1 has an interesting profile due to its inability to use all of the carbon sources of the API 50CHB system except aesculin.

### 3.3. ITS-PCR Fingerprinting Assay

**Figure 2:**
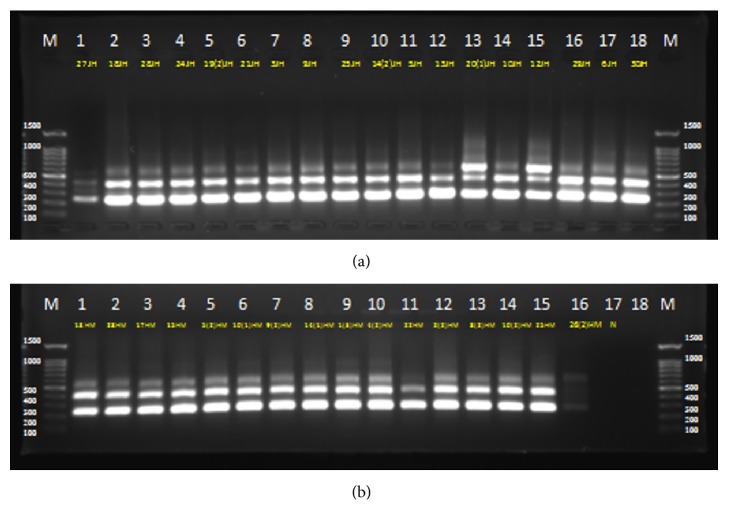
Amplification of 16S–23S internal transcribed spacer region using S-D-Bact-1494 and L-D-Bact-0035 primers. Lanes 1–18 in (a) represent samples obtained from Jordan Al-Hemma. Lanes 1–16 in (b) represent samples from Maain and Zara hot springs; Lane 17 represent a negative control. M; molecular marker.

ITS-PCR products were analyzed by electrophoresis through 1% agarose gels. Several of the bands had low migration rates and remained in the upper part of the gel at apparent positions above the position of the 600 bp standard.  Figure 2 shows the amplification of 16S–23S internal transcribed spacer region using primers S-D-Bact-1494-a-S-20 and L-D-Bact-0035-a-A-15. ITS-PCR experiments were repeated three times using different DNA extractions for amplification. Electrophoretic analysis of the amplified products consistently showed two to four intense, sharp bands (major fragments) for each sample, ranging in size from 300 to 600 bp. On the basis of band difference of major bands, isolates were divided into 10 haplotypes.  Figure 2 shows the ten representatives ITS- PCR patterns of the samples.

### 3.4. Molecular Identification of the Isolates

The final identification and phylogenetic analysis of the isolates was assessed by the 16S rDNA sequencing. The 16S rDNA sequences from the ten haplotypes were aligned with their closely related reference bacterial sequences obtained from the GenBank by Basic Local Alignment Search Tool (BLAST) program. Sequence analysis showed high similarity with those of the reference strains available in the GenBank databases. The 16S rDNA sequence alignment revealed that the isolates H2–H10 fell within the species* Bacillus licheniformis* with moderate sequence similarity, indicating that they may be potential new subspecies. However, sequence analysis showed a strong similarity (99%) between the test strain H1 and the representative strains in gene bank of* Thermomonas hydrothermalis*.

**Figure 3:**
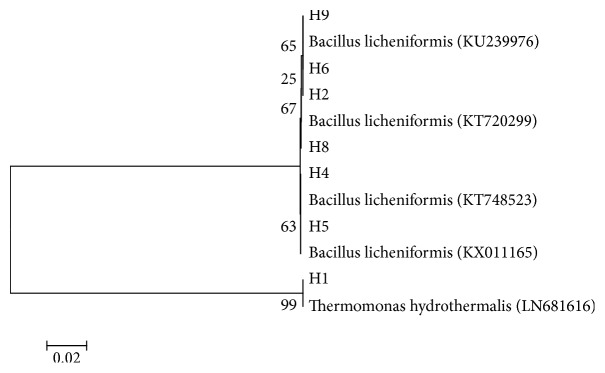
Phylogenetic tree based on total 16S rDNA sequencing (software MEGA 6.0).

The combination of the ITS-PCR fingerprinting and 16S rDNA gene sequencing techniques allowed the identification of ten isolates to the species level. The results revealed a clear domination of the genus* Bacillus* represented by* B. licheniformis* between different hot springs covering a wide geographic area in Jordan. Based upon 16S rDNA sequence alignment, phylogenetic tree was constructed for all the isolated strains (Figure 3). The sequence of H1 isolate showed similarity with* Thermomonas hydrothermalis *(99%), while the H2 to H10 profiles correspond to* B. licheniformis*.

### 3.5. Production of Extracellular Enzymes

**Figure 4:**
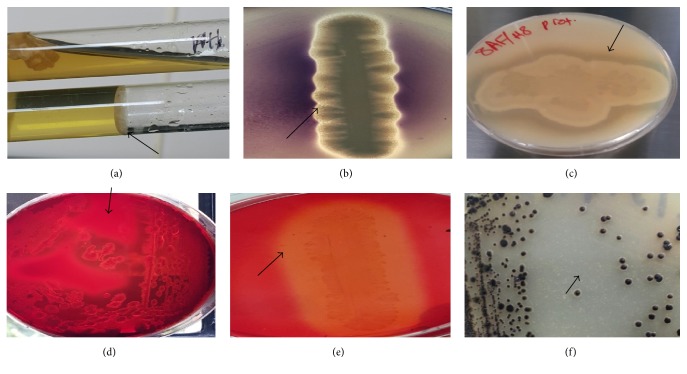
Enzyme activity of* Bacillus* species isolated from Jordanian hot springs. (a) Gelatinase activity, (b) amylase activity, (c) protease activity, (d) beta-hemolytic activity, (e) cellulase activity, and (f) lecithinase activity. Black arrows represent enzyme action.

**Figure 5:**
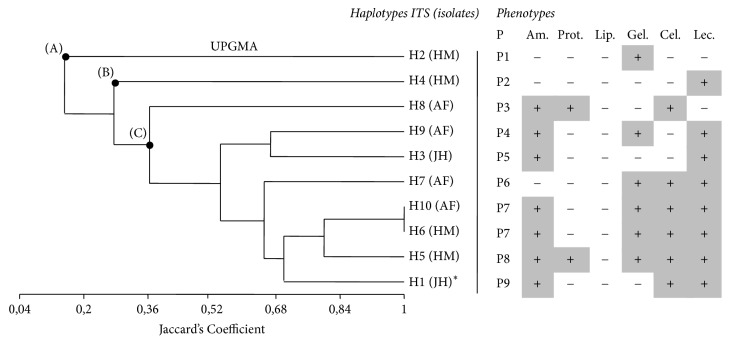
Dendrogram of enzymatic characteristics of ten bacterial strains isolated from hot springs, constructed based on UPGMA cluster analysis and using the Jaccard similarity coefficient. Am.: amylase; Prot: protease; Lip: lipase; Gel: gelatinase; Cel: cellulase; Lec: lecithinase; P: phenotype. ^*^Different strain than* Bacillus*.

Bacterial isolates collected from hot springs were screened for amylase, protease, lipase, gelatinase, cellulose, and lecithinase activity (Figure 4). Among the ten identified haplotypes, at least one extracellular hydrolytic enzyme was produced by each isolate. Seven isolates (70%) produced amylases, 2 isolates (20%) produced proteases, 6 isolates (60%) produced gelatinase, 6 isolates (60%) produced cellulose, and 8 isolates (80%) produced lecithinase enzyme. No lipase activity was observed among the isolates. In addition, 1 isolate (10%) combined five of the tested enzymes, 4 isolates (40%) produced three extracellular enzymes, 1 (10%) isolate produced two enzymes, and 2 isolates produced one enzyme screened (20%) (Table 4). SPSS correlation between enzymes activity and various biochemical tests used for characterization of the ten haplotypes was estimated statistically (Table 5). For further clarification of enzymatic activity, a dendrogram from enzymatic profiles of H1–H10 isolates was constructed using the MVSP v3.2 software, based on UPGMA method and Jaccard coefficient (Figure 5).

## 4. Discussion

Thermal springs represent extreme niches whose pristine quality is maintained over a period of time. The terrestrial hot springs that exist on earth [35] represent hot spots for unusual forms of life, genes, and metabolites. Ever since Thomas Brock discovered the presence of* Thermus aquaticus* in the thermal vents of Yellowstone National Park, a number of researchers have investigated similar environments all over the world. The earth we are existing on is filled with variety of microorganisms that researchers are still far away from being able to complete their identification and isolation, this lead to intensive and extended researches to be carried out in order to fully investigate such promising microorganisms.

Worldwide, geothermal areas which are favorable habitats for thermophilic organisms are limited to a restrict number of sites. In Jordan, there are several hot springs renowned for their rejuvenating and medicinal qualities. The temperatures are often higher than 40°C. In these conditions living organisms have to cope with extremes temperature, low humidity, and low availability of nutritional compounds. These conditions reduce biodiversity but some bacteria developed survival strategies in order to adapt to such stress. In this study, a total of ten bacterial strains isolated from different hot springs were encoded in the form of H1–H10 and then subjected to various morphological, biochemical, molecular, and physiological tests. All isolates except H1 were Gram-positive with spore-forming rod-shaped morphology (Figure 1). Strains were able to grow at a temperature of 50°C. Therefore, they could be classified as thermophilic bacteria according to Brock [36], Perry and Staley [37], and Souza and Martins [38]. Morphological and microscopic characteristics for nine of the isolated strains were similar to the characteristics of the genus* Bacillus*, as was described by Gordon et al. [39] and Souza and Martins [38].

The genus* Bacillus* was isolated from all explored sites; the presence of* Bacillus* in all sampled locations could be due to the ability of this genus to move at high rates and their resistance to harsh environmental conditions [40], in addition to its adaptation for hot surroundings [22, 41]. Strains of* Bacillus* have been dominant in studies carried out by different researchers cross the world. For example, 97.5% of strains recovered by Aanniz et al. [22] from Moroccan hot springs were belonging to this genus. Moreover, Maugeri et al. [2] isolated 87 thermophilic, aerobic, and spore-forming bacteria from Aeolian Islands (Italy), which were the dominant species that were retained to genus* Bacillus*. In addition, thermophilic* Bacillus *was the dominant strain from Jordanian hot springs as reported by Abou-Shanab [42] and Malkawi and Al-Omari [24].

In the present study, the results obtained from ITS-PCR and 16S rDNA for the ten isolates were analyzed by BLAST. Based on BLAST alignment of these isolates to GenBank sequence, the phylogenetic tree was constructed (Figure 3). This allows identification of the ten isolates to the species level. Among these isolates, nine of them (H2–H10) belonged to the species* B. licheniformis* with moderate sequence similarity (25–67%), and one of the isolates showed strong similarity (99%) to the genus* Thermomonas hydrothermalis*. The phylogenetic tree derived from 16S rDNA, in addition to the difference in the morphological and physiological behavior of the strains (H2–H10) (Tables 2 and 3), revealed the possibility of the presence of subspecies among* B. licheniformis*. Variant subspecies genotypes are terms expressing the concept of microheterogeneity within a species. The significance of subspecies seems to allow the possibility of distinguishing important phenotype and niche differences between the isolates [43]. However, the traditional methods including morphological and biochemical characterization used for identification of the isolates are troublesome, and sometimes the results are difficult to interpret. Information obtained by sequencing make it possible to identify the 10 haplotypes to species level.

These findings are consistent with other studies carried out by De Clerck and De Vos [44]; Burgess et al. [45]; and Aanniz et al. [22]. Furthermore, Manachini et al. [46] reported three distinct groups among 182* B. licheniformis* strains isolated. Similarly, this strain has also been isolated from different hot springs worldwide, such as India [19–47], Morocco [48], Turkey [4], Bulgari [49], and Indonesia [50]. In Jordan, previous findings reported the isolation of thermophilic bacteria belonging to the genus Bacillus. For instance, a thermostable protease producing* Bacillus pumilus* has been isolated from Mae'en hot springs in the north of Jordan valley [51]. Moreover,* Geobacillus* species were isolated and characterized by Obeidat et al. [52] to determine their enzymatic activities.

It was clearly observed that all Jordanian hot springs investigated in this study were found to be populated with* Bacillus licheniformis* species. The low diversity observed in our study may be correlated to the environmental conditions such as high temperature and the nutritional status available for the growth of* Bacillus* in water strata of the hot springs. Previous studies on thermal springs have shown an increasing diversity with decreasing temperature [53]. The microbial diversity of the five hot springs in Jordan was studied by Malkawi and Al-Omari [24] using both microbiological and molecular approaches. Most of the isolated bacteria reported were Gram-positive rods (94.7%) and (90.9%) belong to the genus* Bacillus*.

Ecological environment established on the hot springs was long-known to be moderate to high temperature and nutrient-poor. These waters, springing out from below the ground, form an isolated environment from a biogeographic point of view. Strains of the genus* Bacillus* are well adapted to hot environments. They have also generally simple nutritional needs. Therefore, they are able to colonize oligotrophic niches like salt marshes, hot springs, and desert soils [54].

On the other hand, the interesting finding in this study, is the isolation of strain H1, which was identified by 16S rDNA as* Thermomonas hydrothermalis*; there are no previous reports about the isolation of this strain from hot springs in Jordan; additionally, worldwide few reports concerning this strain are available. The genus* Thermomonas*, belonging to the family Xanthomonadaceae was firstly established and described by Busse et al. [55]. This genus comprises five species isolated from a wide range of habitats, kaolin slurry [the type species,* Thermomonas haemolytica* [55]], a hot spring [*Thermomonas hydrothermalis *[56]], a denitrification reactor [*Thermomonas brevis* and* Thermomonas fusca* [57]], and a ginseng field [*Thermomonas koreensis *[58]].* Thermomonas hydrothermalis* was isolated for the first time from hot springs at São Gemil in central Portugal [56]. It was closely related to* Thermomonas haemolytica* but has a higher growth temperature range than this species. This strain was described as Gram-negative, rod-shaped cells. Cells are nonmotile, and they form light brown colored colonies 0.5 to 2.0 mm in diameter and a diffusible brown pigment in older cultures. Recently, this slightly thermophilic bacterium was isolated from geothermal areas in Turkey [4]. Since there are not many reports regarding these isolates, this makes it a fascinating bacterial strain to be further investigated for its biotechnological and environmental applications.

One of the important potentials of thermophilic microorganisms is their enzymatic activities; due to their ability to function under sever conditions such as temperature, pH, and pressure, this will intensify their importance in industrial and biotechnological areas. Such enzymes can be isolated from extremophilic microorganisms. This indicated that the strains may have developed special mechanisms genetically and physiologically to be able to utilize available organic matter, via enzyme production [48], in addition to the possibility of adopting system by these microorganisms to survive in such conditions and uptake of any available nutrients. Microorganisms that possess thermostable enzymes have many attractive features such as their ability to minimize the possibility of microbial contamination in large-scale industrial reactions and working for long durations [59, 60].

In this study, none of the ten isolates was lipase positive, while six of them were cellulase positive (including H1), and two of them only were protease positive. The constructed UPGMA dendrogram of the enzymatic characteristics of ten bacterial strains isolated from five hot springs showed three clusters (A, B, and C). The first cluster (A) included one isolate corresponding to phenotype P1 and haplotype ITS (H2). The second cluster (B) contained also one isolated strain representing the phenotype P2. The last cluster (C) could be subdivided into seven branches corresponding to 6 different phenotypes (P3 to P9). The phenotypes P3, P4, P5, P6, P8, and P9 were represented, respectively, by the isolates of haplotypes ITS H8, H9, H3, H7, H5, and H1. The phenotype P7 regrouped two isolates of two different haplotypes ITS (H6 and H9). Based on the dendrogram obtained results, the ten studied isolates exhibited a high phenotypic diversity.

A positive correlation was observed by H5 and H8, linking protease production with their abilities to utilize arabinose, ribose, xylose, sorbose, mannitol, mannoside, starch, glycogen, and fucose (*P* < 0.05). However, cellulase, lecithinase, and amylase produced by* B. licheniformis strains and T. hydrothermalis* showed a negative correlation (*P* < 0.05) with some biochemical tests indicating that isolates with high cellulase, lecithinase, and amylase activity have low capability to metabolize arabinose, ribose, xylose, mannitol, mannoside, starch, glycogen, and fucose, and vice versa.

*Bacillus* strains have been studied intensively for its capability to produce very important thermoenzymes [61]. As it was observed the variation of the excretion of extracellular enzymes among strains (H2–H10) which confirms the presence of subspecies of* Bacillus licheniformis*. Results obtained are consistent with other reports about the amylolytic, proteolytic, and cellulatic activity [22, 62]. However, there were not previous detailed enzyme profiles available for* Thermomonas* strains except a study by Baltaci et al. [4], which reported positive amylase and lipase activity and negative cellulase, which is contrary to the results obtained in our study, where the strain showed positive cellulase activity but negative lipase activity. This encourages further studies to carry on further enzymatic analysis with this strain in the future.

## 5. Conclusions

The thermophilic bacteria* Bacillus licheniformis* and* Thermomonas hydrothermalis *were isolated and their preliminary enzymatic potential was characterized. This is the first report on isolation of a* Thermomonas *strain from Jordanian hot springs. The diversity in phenotypic and enzymatic analysis among* B. licheniformis* strains indicated the presence of subspecies. These promising results can be exploited further for production of biotechnological important and industrially thermostable enzymes. This study widens the opportunities for further research to be conducted to explore more the immense significance of these strains especially* Thermomonas *isolates, where there is lack of intensive studies regarding this organism.

## Figures and Tables

**Table 1:** Physicochemical properties of the hot springs.

Location	Temperature (C°)	PH	Conductivity (ms/cm)	Haplotype
Al- Hemma	40–56	7.08–8.41	1.06–1.41	H1, H3
Hammamat Afra & Al-Burbita	39.9–47.4	7.03–8.03	0.51–0.76	H7, H8, H9, H10
Hammamat Ma'in & Zara	43–60	7.5–8.6	2.12–3.27	H2, H4, H5, H6

**Table 2:** Colony morphology of the ten haplotypes isolated from Jordanian hot springs.

Samples	Hemolysis	Light transmission	Consistency	Shape	Margin	Elevation	Color
1JH	*β*-Hemolysis	Opaque	Rough	irregular	Erose	Flat	Gray
2HM	*γ*-Hemolysis	Translucent	Smooth	irregular	Umbonate	Flat	White
3JH	*α*-Hemolysis	Translucent	Rough	irregular	Finger-like projection	Flat	White-creamy
4HM	*γ*-Hemolysis	Translucent	Rough, wrinkled	Irregular	Undulate	Raised	White-light red
5HM	*γ*-Hemolysis	Translucent	Smooth, mucous	irregular	Lobate	Flat	White- yellow
6HM	*α*-Hemolysis	Opaque	Rough, wrinkled	Irregular	Erose	Flat	Gray-yellow
7AF	*γ*-Hemolysis	Translucent	Smooth	regular	Undulate	Flat	White-creamy
8AF	*γ*-Hemolysis	Translucent	Mucous, Smooth	regular	Erose	Raised	Gray-brown
9AF	*α*-Hemolysis	Opaque	Smooth	round	Entire	Raised	White- creamy,
10AF	*α*-Hemolysis	Opaque	Rough	irregular	Undulate	Flat	White-gray

**Table 3:** Carbohydrate utilization profile using API 50CH for the ten isolates.

Substrate	H1	H2	H3	H4	H5	H6	H7	H8	H9	H10
Glycerol	−	−	−	+	−	−	−	−	−	−
Erythritol	−	−	−	+	−	−	−	+	−	−
D-Arabinose	−	−	−	+	−	−	−	−	−	−
L-Arabinose	−	+	+	+	+	−	−	−	+	+
Ribose	−	+	+	+	−	−	−	+	+	+
D-Xylose	−	+	+	+	−	+	+	+	+	+
L-Xylose	−	−	−	−	−	−	−	+	−	−
Adonitol	−	−	−	−	−	−	−	−	−	−
B-Methyl xyloside	−	−	−	−	−	−	−	−	−	−
Galactose	−	+	+	−	−	−	−	−	−	−
Glucose	−	+	+	+	+	+	+	+	+	+
Fructose	−	+	+	+	+	+	+	+	+	+
Mannose	−	+	+	+	+	−	−	+	+	+
L-Sorbose	−	−	−	−	−	−	−	+	−	−
Rhamnose	−	−	−	−	−	−	−	−	−	−
Dulcitol	−	−	−	−	−	−	−	−	−	−
Inositol	−	+	+	−	+	−	−	−	+	+
Sorbitol	−	+	+	+	−	+	+	+	+	+
Mannitol	−	−	−	−	−	−	−	+	−	−
L-Methyl-D-mannoside	−	−	−	−	−	−	−	+	−	−
D-Methyl-D-glucoside	−	+	+	+	−	−	−	−	−	−
N-Acetylglucosamine	−	+	+	+	−	−	−	+	−	+
Amygdalin	−	+	−	+	+	−	−	−	−	+
Arbutin	−	−	−	+	+	−	−	+	−	+
Aesculin	+	+	+	+	+	−	−	+	+	+
Salicin	−	−	+	+	+	−	+	+	−	+
D-Cellobiose	−	+	+	+	+	−	−	+	−	+
D-Maltose	−	+	+	+	+	−	−	+	−	+
D-Lactose	−	−	−	−	−	−	−	−	−	−
D-Sucrose	−	−	−	−	−	−	−	−	−	−
Trehalose	−	−	+	+	+	−	−	+	−	+
Gentiobiose	−	−	+	+	+	−	−	+	−	+
Melibiose	−	−	−	−	−	−	−	−	−	−
Raffinose	−	−	−	−	−	−	−	−	−	−
Melezitose	−	−	−	−	−	−	−	−	−	−
Starch	−	−	−	−	−	−	−	+	−	−
Glycogen	−	−	−	−	−	−	−	+	−	−
Inulin	−	−	−	−	−	−	−	−	−	−
D-Turanose	−	−	−	−	−	−	−	−	−	−
D-Tagatose	−	−	−	−	−	−	−	−	−	−
D-Fucose	−	−	−	−	−	−	−	+	−	−
L-fucose	−	−	−	−	−	−	−	−	−	−
D-Lyxose	−	−	−	−	−	−	−	−	−	−
D-Arabitol	−	−	−	−	−	−	−	−	−	−
L-Arabitol	−	−	−	−	−	−	−	−	−	−
Xylitol	−	−	−	−	−	−	−	−	−	−
Gluconate	−	−	−	−	−	−	−	−	−	−
2-Ketogluconate	−	−	−	−	−	−	−	−	−	−
5-Ketogluconate	−	−	−	−	−	−	−	−	−	−

**Table 4:** Variation in enzymatic activity produced by *Thermomonas hydrothermalis* (H1) and *Bacillus licheniformis *(H2–H10) strains. JH, Jordan Hemma; HM, Hammamat Mae'en; AF, Afra.

Haplotypes	Amylase	Protease	Lipase	Gelatinase	Cellulase	Lecithinase
H1 (JH)	**+**	−	−	−	**+**	**+**
H2 (HM)	−	−	−	**+**	−	−
H3 (JH)	+	−	−	−	−	**+**
H4 (HM)	−	−	−	−	−	**+**
H5 (HM)	**+**	**+**	−	**+**	**+**	**+**
H6 (HM)	**+**	−	−	**+**	**+**	**+**
H7 (AF)	−	−	−	**+**	**+**	**+**
H8 (AF)	**+**	**+**	−	−	**+**	−
H9 (AF)	**+**	−	−	**+**	−	**+**
H10 (AF)	**+**	−	−	**+**	**+**	**+**

**Table 5:** Correlation between protease, cellulose, lecithinase, and amylase activity produced by *T. hydrothermalis* and *B. licheniformis* strains and some biochemical tests.

Substrate	Protease PC (*P* value)^**∗**^	Cellulase PC (*P* value)	Lecithinase PC (*P* value)	Amylase PC (*P* value)
L-Arabinose	—	−0.667 (0.035)^*∗*^	—	—
Ribose	—	−0.667 (0.035)	—	—
L-Xylose	0.667 (0.035)	—	—	—
L-Sorbose	0.667 (0.035)	—	—	−0.667 (0.035)
Mannitole	0.667 (0.035)	—	—	−0.667 (0.035)
L-Methyl-D-mannoside	0.667 (0.035)	—	—	−0.667 (0.035)
D-Methyl-D-glucoside	—	—	−0.802 (0.005)	—
Starch	0.667 (0.035)	—	—	−0.667 (0.035)
Glycogen	0.667 (0.035)	—	—	−0.667 (0.035)
D-Fucose	0.667 (0.035)	—	—	−0.667 (0.035)

^*∗*^PC: Pearson's correlation.

## References

[1] Khalil A. (2011). Screening and characterization of thermophilic bacteria (lipase, cellulase and amylase producers) from hot springs in Saudi Arabia. *Journal of Food, Agriculture and Environment*.

[2] Maugeri T. L., Gugliandolo C., Caccamo D., Stackebrandt E. (2001). A polyphasic taxonomic study of thermophilic bacilli from shallow, marine vents. *Systematic and Applied Microbiology*.

[3] Boomer S. M., Noll K. L., Geesey G. G., Dutton B. E. (2009). Formation of multilayered photosynthetic biofilms in an alkaline thermal spring in Yellowstone national Park, Wyoming. *Applied and Environmental Microbiology*.

[4] Baltaci M. O., Genc B., Arslan S., Adiguzel G., Adiguzel A. (2017). Isolation and Characterization of Thermophilic Bacteria from Geothermal Areas in Turkey and Preliminary Research on Biotechnologically Important Enzyme Production. *Geomicrobiology Journal*.

[5] Adiguzel A., Inan K., Sahin F. (2011). Molecular diversity of thermophilic bacteria isolated from Pasinler hot spring (Erzurum, Turkey). *Turkish Journal of Biology*.

[6] Genc B., Nadaroglu H., Adiguzel A., Baltaci O. (2015). Purification and characterization of an extracellular cellulase from *Anoxybacillus gonensis* O9 isolated from geothermal area in Turkey. *Journal of Environmental Biology*.

[7] Tango M. S. A., Islam M. R. (2002). Potential of extremophiles for biotechnological and petroleum applications. *Energy Sources*.

[8] Singh G., Bhalla A., Kaur P., Capalash N., Sharma P. (2011). Laccase from prokaryotes: A new source for an old enzyme. *Reviews in Environmental Science and Biotechnology*.

[9] Bhalla A., Bansal N., Kumar S., Bischoff K. M., Sani R. K. (2013). Improved lignocellulose conversion to biofuels with thermophilic bacteria and thermostable enzymes. *Bioresource Technology*.

[10] Dettmer A., dos Anjos P. S., Gutterres M. (2013). Special review paper: Enzymes in the leather industry. *Journal of the American Leather Chemists Association*.

[11] Chirumamilla R. R., Muralidhar R., Marchant R., Nigam P. (2001). Improving the quality of industrially important enzymes by directed evolution. *Molecular and Cellular Biochemistry*.

[12] Vijayalakshmi S., Venkat Kumar S., Thankamani V. (2011). Optimization and cultural characterization of *Bacillus* RV.B2.90 producing alkalphilicthermophilic protease. *Research Journal of Biotechnology*.

[13] Pandey A., Selvakumar P., Soccol C. R., Nigam P. (1999). Solid state fermentation for the production of industrial enzymes. *Current Science*.

[14] Hardiman E., Gibbs M., Reeves R., Bergquist P. (2010). Directed evolution of a thermophilic *β*-glucosidase for cellulosic bioethanol production. *Applied Biochemistry and Biotechnology*.

[15] Hmidet N., El-Hadj Ali N., Haddar A., Kanoun S., Alya S.-K., Nasri M. (2009). Alkaline proteases and thermostable *α*-amylase co-produced by *Bacillus licheniformis* NH1: characterization and potential application as detergent additive. *Biochemical Engineering Journal*.

[16] Sathya R., Ushadevi T. (2014). Industrially important enzymes producing streptomyces species from mangrove sediments. *International Journal of Pharmacy and Pharmaceutical Sciences*.

[17] Gupta G., Srivastava S., Khare S. K., Prakash V. (2014). Extremophiles: an overview of microorganism from extreme environment. *International Journal of Agriculture, Environment and Biotechnology*.

[18] Busk P. K., Lange L. (2013). Cellulolytic Potential of Thermophilic Species from Four Fungal Orders. *AMB Express*.

[19] Kumar M., Yadav A. N., Tiwari R., Prasanna R., Saxena A. K. (2014). Deciphering the diversity of culturable thermotolerant bacteria from Manikaran hot springs. *Annals of Microbiology*.

[20] Yoneda Y., Yoshida T., Yasuda H., Imada C., Sako Y. (2013). A thermophilic, hydrogenogenic and carboxydotrophic bacterium, Calderihabitans maritimus gen. nov., sp. nov., from a marine sediment core of an undersea caldera. *International Journal of Systematic and Evolutionary Microbiology*.

[21] Cihan A. C., Cokmus C., Koc M., Ozcan B. (2014). Anoxybacillus calidus sp. nov., a thermophilic bacterium isolated from soil near a thermal power plant. *International Journal of Systematic and Evolutionary Microbiology*.

[22] Aanniz T., Ouadghiri M., Melloul M. (2015). Thermophilic bacteria in Moroccan hot springs, salt marshes and desert soils. *Brazilian Journal of Microbiology*.

[23] Swarieh A. (2000). Geothermal energy resources in Jordan, country update report. *Proceedings World Geothermal Congress*.

[24] Malkawi H. I., Al-Omari M. N. (2010). Culture-dependent and culture-independent approaches to study the bacterial and archaeal diversity from jordanian hot springs. *African Journal of Microbiology Research*.

[25] Daffonchio D., Cherif A., Borin S. (2000). Homoduplex and heteroduplex polymorphisms of the amplified ribosomal 16S-23S internal transcribed spacers describe genetic relationships in the “*Bacillus cereus* group”. *Applied and Environmental Microbiology*.

[26] Sambrook J., Russell D. W., Sambrook J., Russell D. W. (2001). Rapid isolation of yeast DNA. *Molecular Cloning: A Laboratory Manual*.

[27] Prescott L. M., Harley G. P., Klein D. E. (1993). *Microbiology*.

[28] Ray A. K., Bairagi A., Sarkar Ghosh K., Sen S. K. (2007). Optimization of fermentation conditions for cellulase production by Bacillus subtilis CY5 and Bacillus circulans TP3 isolated from fish gut. *Acta Ichthyologica et Piscatoria*.

[29] Shaikh N. M., Patel A. A., Mehta S. A., Patel N. D. (2013). Isolation and screening of cellulolytic bacteria inhabiting different environment and optimization of cellulose production. *Universal Journal of Environmental Researcg and Technology*.

[30] Nord C.-E., Sjöberg L., Wadström T., Wretlind B. (1975). Characterization of three Aeromonas and nine Pseudomonas species by extracellular enzymes and haemolysins. *Medical Microbiology and Immunology*.

[31] Rollof J., Hedstrom S. A., Nilsson‐Ehle P. (1987). Lipolytic activity of staphylococcus aureus strains from disseminated and localized infections. *Acta Pathologica Microbiologica Scandinavica Series B: Microbiology*.

[32] Burke V., Robinson J. O., Richardson C. J. L., Bundell C. S. (1991). Longitudinal studies of virulence factors of pseudomonas aeruginosa in cystic fibrosis. *Pathology*.

[33] Betty A. F., Daniel L. S., Weissfeld A. S. (2007). *Bailey & Scott's Diagnostic Microbiology*.

[34] Cowan D. A. (1991). Industrial enzymes. *Biotechnology, the Science and the Business*.

[35] Tobler D. J., Benning L. G. (2011). Bacterial diversity in five Icelandic geothermal waters: Temperature and sinter growth rate effects. *Extremophiles*.

[36] Brock T. D. (1978). *Thermophilic Microorganisms and Life at High Temperatures*.

[37] Perry I. I., Staley I. T., Perry II., Staley IT. (1997). Taxonomy of eubacteria and archaea. *Microbiology: Diversity and Dynamics*.

[38] Souza A. N., Martins M. L. (2001). Isolation, properties and kinetics of growth of a thermophilic *Bacillus*. *Brazilian Journal of Microbiology*.

[39] Gordon R. E., Haynes W. C., Pang H. N. (1973). *The Genus Bacillus. Agricultural Research Service, United State, Department of Agriculture*.

[40] Connor N., Sikorski J., Rooney A. P. (2010). Ecology of speciation in the genus Bacillus. *Applied and Environmental Microbiology*.

[41] Kawasaki Y., Aoki M., Makino Y. (2012). Characterization of moderately thermophilic bacteria isolated from saline hot spring in Japan. *Microbiology Indonesia*.

[42] Abou-Shanab R. A. I. (2007). Characterization and 16S rDNA identification of thermo-tolerant bacteria isolated from hot springs. *Journal of Applied Sciences Research*.

[43] Chen H. I., Hulten K., Clarridge J. E. (2002). Taxonomic subgroups of Pasteurella multocida correlate with clinical presentation. *Journal of Clinical Microbiology*.

[44] De Clerck E., De Vos P. (2004). Genotypic diversity among Bacillus licheniformis strains from various sources. *FEMS Microbiology Letters*.

[45] Burgess S. A., Lindsay D., Flint S. H. (2010). Thermophilic bacilli and their importance in dairy processing. *International Journal of Food Microbiology*.

[46] Manachini P. L., Fortina M. G., Levati L., Parini C. (1998). Contribution to phenotypic and genotypic characterization of Bacillus licheniformis and description of new genomovars. *Systematic and Applied Microbiology*.

[47] Verma A., Gupta M., Shirkot P. (2014). Isolation and characterization of thermophilic bacteria in natural hot water springs of himachal Pradesh (india). *The Bioscan*.

[48] Berrada I., Willems A., De Vos P. (2012). Diversity of culturable moderately halophilic and halotolerant bacteria in a marsh and two salterns a protected ecosystem of lower loukkos (Morocco). *African Journal of Microbiology Research*.

[49] Derekova A., Mandeva R., Kambourova M. (2008). Phylogenetic diversity of thermophilic carbohydrate degrading bacilli from Bulgarian hot springs. *World Journal of Microbiology and Biotechnology*.

[50] Ibrahim D., Zhu H. L., Yusof N., Sheng Hong L. (2013). *Bacillus licheniformis* BT5.9 isolated from changar hot spring, malang, Indonesia, as a potential producer of thermostable *α*-amylase. *Tropical Life Science Research*.

[51] Al-Qodah Z., Daghistani H., Alananbeh K. (2013). Isolation and characterization of thermostable protease producing *Bacillus pumilus* from thermal spring in Jordan. *African Journal of Microbiology Research*.

[52] Obeidat M., Khyami-Horani H., Al-Zoubi A., Otri I. (2012). Isolation, characterization, and hydrolytic activities *Geobacillus* species from Jordanian hot springs. *African Journal of Biotechnology*.

[53] Sompong U., Hawkins P. R., Besley C., Peerapornpisal Y. (2005). The distribution of cyanobacteria across physical and chemical gradients in hot springs in northern Thailand. *FEMS Microbiology Ecology*.

[54] Khiyami M. A., Serour E. A., Shehata M. M., Bahklia A. H. (2012). Thermo-aerobic bacteria from geothermal springs in Saudi Arabia. *African Journal of Biotechnology*.

[55] Busse H.-J., Kämpfer P., Moore E. R. B. (2002). Thermomonas haemolytica gen. nov., sp. nov., a *γ*-proteobacterium from kaolin slurry. *International Journal of Systematic and Evolutionary Microbiology*.

[56] Alves M. P., Rainey F. A., Fernanda Nobre M., da Costa M. S. (2003). *Thermomonas hydrothermalis* sp. nov., a new slightly thermophilic *γ*-proteobacterium isolated from a hot spring in central Portugal. *Systematic and Applied Microbiology*.

[57] Mergaert J., Cnockaert M. C., Swings J. (2003). Thermomonas fusca sp. nov. and Thermomonas brevis sp. nov., two mesophilic species isolated from a denitrification reactor with poly(E-caprolactone) plastic granules as fixed bed, and emended description of the genus Thermomonas. *International Journal of Systematic and Evolutionary Microbiology*.

[58] Kim M. K., Im W.-T., In J.-G., Kim S.-H., Yang D.-C. (2006). Thermomonas koreensis sp. nov., a mesophilic bacterium isolated from a ginseng field. *International Journal of Systematic and Evolutionary Microbiology*.

[59] Wati L., Dhamija S. S., Singh D., Nigam P., Marchant R. (1996). Characterisation of genetic control of thermotolerance in mutants of *saccharomyces cerevisiae*. *Journal of Genetic Engineer and Biotechnologist*.

[60] Wang X., Li D., Watanabe T. (2012). A glucose/o-2 biofuel cell using recombinant thermophilic enzymes. *International Journal of Electrochemical Science*.

[61] Meintanis C., Chalkou K. I., Kormas K. A. (2008). Application of rpoB sequence similarity analysis, REP-PCR and BOX-PCR for the differentiation of species within the genus Geobacillus. *Letters in Applied Microbiology*.

[62] Popović M. K., Senz M., Bader J., Skelac L., Schilf W., Bajpai R. (2014). Positive effect of reduced aeration rate on secretion of alpha-amylase and neutral proteases during pressurised fermentation of thermophilic Bacillus caldolyticus. *New Biotechnology*.

